# Safety Risks of Plant Fiber/Plastic Composites (PPCs) Intended for Food Contact: A Review of Potential Hazards and Risk Management Measures

**DOI:** 10.3390/toxics9120343

**Published:** 2021-12-09

**Authors:** Hong Zhang, Yunxuan Weng

**Affiliations:** 1 School of Light Industry, Beijing Technology and Business University, Beijing 100048, China; zhanghong@cfsa.net.cn; 2 China National Center for Food Safety Risk Assessment, Beijing 100022, China; 3 College of Chemistry and Materials Engineering, Beijing Technology and Business University, Beijing 100048, China; 4 Beijing Key Laboratory of Plastic Hygiene and Safety Quality Evaluation Technology, Beijing 100048, China

**Keywords:** safety risk, plant fiber/plastic composite, food contact materials, potential hazards

## Abstract

Plant fiber/plastic composites (PPCs), with the benefits of low cost and easy processing, have been widely used in the production of various food contact products. They are generally considered to be economical and environmentally friendly because of their natural raw materials (plant fibers) and recommended to be one of the ideal alternatives to traditional petrochemical-based plastics. However, in addition to plastic resins and plant fibers, some indispensable additives are involved in the production process of PPCs, which may pose food safety risks. To date, excessive migration of hazardous substances (such as melamine) has been reported in some products made of PPCs, and the safety and applicability of PPCs as food contact materials need to be further studied. In this paper, the main raw materials of PPCs used for food contact are taken as the pointcut to analyze the possible hazards, sources of hazards, and existing risk management measures in various countries. The conclusion shows that PPCs used for food contact may have potential safety risks at present. However, systematic research on migration methods and safety assessment are still insufficient, and further studies are needed regarding the main safety risks and migration patterns.

## 1. Introduction

In the context of circular economy and sustainable development, countries all over the world are looking for new materials which are more environmentally friendly and economical, to replace traditional petrochemical-based plastics. Bio-based plastics refer to a kind of polymeric material with plastic characteristics produced from biomass [1,2] such as cellulose, starch, fiber, and protein. Most bio-based plastics are degradable, and the development of bio-based plastics meets the developmental requirements of energy conservation, environmental protection, and circular economy [3,4]. In China, industrial restructuring proposals to encourage the development of bio-based materials have been put forward, with an estimated economic impact of about 30 trillion yuan [5].

As a kind of bio-based material, plant fiber, which is a renewable resource, has the characteristics of low cost, easy processing, low density, and biodegradability [6]. It is suitable for blending with starch, chitin, or other degradable materials to produce degradable food contact materials [7]. At present, the PPCs made from natural plant fibers such as bamboo, straw, rice husk, bagasse, coffee grounds, and synthetic resins have been widely used in the production of tableware.

However, food contact materials and products produced by blending nature plant fibers with synthetic resins may also have certain safety risks. Relevant studies have shown the migration of various substances in this kind of material, including components derived from plant fibers, synthetic resins, or additives [8]. For example, tableware made of bamboo fiber blended with melamine-formaldehyde resin (MF) has been repeatedly found to have excessive migration of melamine or formaldehyde [9,10].

**Figure 1:**
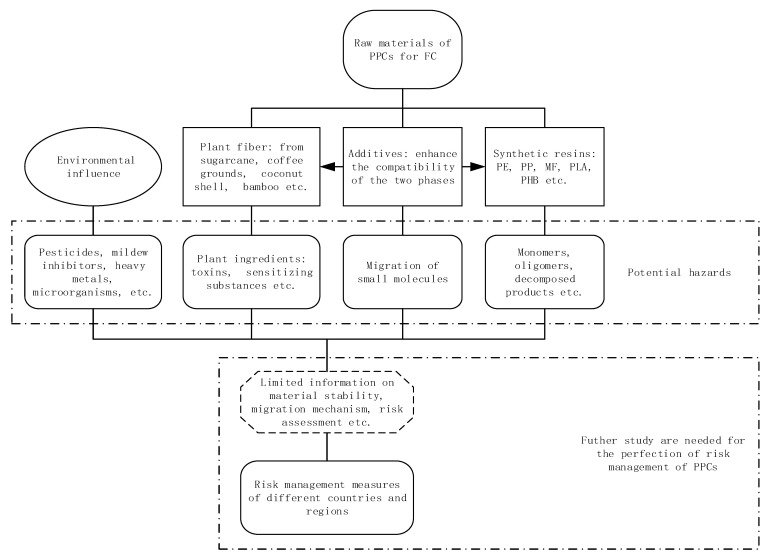
Potential hazards in plastic/plant-fiber composites (PPCs) for food contact may come from raw materials such as plant fibers, synthetic resins and additives, or from environmental influences. At present, several countries and regions have established standards or regulations for the safety management of PPCs.

Here, we review the potential safety risks of food contact materials made of plant fibers and synthetic resins, identify the possible hazards and sources of hazards, analyze risk management measures in some countries and regions, and discuss the current status and the possible trends of safety research (Figure 1).

## 2. Product Types and Main Raw Materials

### 2.1. Product Types

#### 2.1.1. Classification According to the Proportion of Ingredients

According to the different proportions of main raw materials and processing technology, food contact products made of PPCs can be divided into two categories (Table 1). The first one is made of plant fibers (or powders) as the main raw material (up to 90%), with a small amount of resins and other additives [11], and processed by dry molding or wet compression molding process. This kind of material has poor heat and water resistance, and generally has a relatively fast deterioration rate [7]. Therefore, it is difficult to use them repeatedly for a long time. In order to enhance products’ waterproof and oil-proof performance, coating of melamine resin or other materials usually be applied after molding [12,13,14]. The second one uses plastic resins as the main raw material and plant fibers (or powders) as a filler (30~70%), which are usually processed and molded by extrusion molding. These kinds of products are usually made reusable [15,16,17,18,19], and can keep good performance on the premise of lower prices [7]. Considering the differences in composition and application scenarios, the above two types of products should also be treated differently in risk analysis and safety assessment.

#### 2.1.2. Classification According to Degradation Performance

Due to the degradable properties of plant fibers, the degradability of PPCs primarily depends on materials other than plant fibers in the formulation [20]. Therefore, in terms of degradability, PPC food contact materials can be divided into degradable materials and non-degradable materials according to the degradation performance of raw materials. Only PPC products produced by blending with degradable materials, such as starch, polylactic acid (PLA), or poly (butyleneadipate-co-terephthalate) (PBAT) can be regarded as degradable. Composites blended with traditional plastics cannot be completely degraded. At present, although PPCs have the advantage of using plant fibers as renewable resources, the mechanical properties, processability, and water vapor/oxygen barrier properties of PPCs are still inadequate compared with traditional plastics [4,21].

### 2.2. Main Raw Materials

The main raw materials of PPCs include plant fibers, synthetic resins, and additives. Plant fibers are also used as filling agents (additives) in certain materials, while in this paper they were discussed as basic materials other than additives. As the intentionally or non-intentionally added substances in the raw materials are the major components of migration, the types and characteristics of raw materials are important factors that affect the safety of PPCs.

#### 2.2.1. Basic Materials

##### Plant Fiber

The chemical constitution of natural fiber is very complex, including cellulose, hemicellulose, lignin, pectin, wax, pigment, and other substances [6]. Nowadays, many kinds of plant fibers are used to produce PPCs intended for food contact, including bagasse, olive pomace, jute, coconut shell, coffee pomace, bamboo fiber, and other varieties [6]. Due to the difference in plant distribution, the types of plant fibers studied and applied also show certain regional characteristics.

Compared to synthetic fibers, plant fibers of the same quality have stronger mechanical properties [22]. Nevertheless, the disadvantages are their higher hydrophilicity and relatively poor thermal stability [23]. The mechanical properties, hydrophilicity, and thermal stability of plant fibers are related to the composition ratio of cellulose, hemicellulose, and lignin. Generally speaking, the mechanical strength and thermal stability of cellulose that has a higher molecular weight are stronger than those of hemicellulose and lignin, while hemicellulose has stronger water absorptivity [24]. In addition, the quality of plant fiber is unstable, which is easily affected by factors such as regions, seasons, or suppliers [25]. This is also an important factor that limits its application.

##### Synthetic Resin

Synthetic resin is mostly used as a connecting phase or coating in PPCs, which has adhesive, waterproof, and oil-proof properties [7]. All kinds of synthetic resins can be used in the production of PPCs. Traditional petroleum resins such as polypropylene (PP) [26], polyethylene (PE) [27], and MF [11,12], and other biodegradable resin materials such as PLA [3], poly (butylene succinate) (PBS) [27], hydroxybutyrate-co-hydroxyvalerate (PHBV) [28,29], polyhydroxybutyrate (PHB) [21] are the most commonly used resins.

Because of their molecular structure and chemical bond characteristics, degradable resins usually have low stability and heat resistance, therefore with a poor machining performance compared with non-degradable resins [2]. For example, PLA, which is widely used in food contact products, has a fast crystallization rate, low breaking elongation, and poor toughness [30]. As a kind of polyhydroxyalkanoate, PHB resin has high hardness but poor plasticity, which makes it easy to fracture during processing [31,32]. Most degradable resins need modification, blending with other resins or adding filling agents to improve their processability [33,34].

##### Interaction between Plant Fiber and Synthetic Resin

Adding plant fiber into resins can enhance the strength of the composite [35], but will reduce the thermal stability, water vapor permeability, and oxygen resistance of the composite to a certain extent [29,36,37]. Coffee jar lids made from 40% banana fiber with equal HDPE and PLA showed a better impact resistance than pure PLA products [38]. E.L. Sánchez-Safont et al. [21] blended 10–20% almond shell, rice husk, and seaweed with PHB. It was found that, on the one hand, natural plant fibers could enhance the elastic modulus of PHB without affecting its crystallization and degradation properties. On the other hand, compared with pure PHB materials, the barrier properties and thermal stabilities of the composites were reduced to varying degrees. The study of bio-composites made by melt extrusion of coffee silverskin and PHBV showed that the increase of coffee silverskin content could improve the crystallinity, hardness, and heat deflection temperature of the bio-composites [39].

There may be significant differences in physical and chemical properties of plant fibers of different kinds, different producing areas, and different processing methods, thus affecting the performance of the final composites [40]. By comparing water-resisting properties of films made by mixing cellulose and fiber extracted from bagasse and coconut shells according to different proportions, it was found that, compared with pure cellulose films, materials with a small amount of fiber (75% cellulose and 25% fiber) have better water-resisting properties [41]. The olive pomace is a solid waste in the olive oil pressing process, which is rich in lignocellulose. Lammi et al. [42] dried, ground and processed olive pomace into three different fillers, which were then added to PP and PHBV to prepare composites with 5–30% of olive pomace content. The results showed that olive pomace with higher lignin content and weaker polarity could better retain the mechanical properties of PP and PHBV. In contrast, the olive pomace with high cellulose content and strong polarity, as well as the roughly processed olive pomace obviously reduced the mechanical properties and water vapor permeability of composites but had little effect on the oxygen permeability of the materials. The above effects become more significant with the increase of olive pomace content.

Blending synthetic resins with natural plant fibers also has positive economic and social benefits. On the one hand, this method can reduce the use of petrochemical raw materials, promote the utilization of natural resources and reduce carbon emissions. On the other hand, it can also reduce the production cost [4,43], which is beneficial to industrial production and expansion of application scope. Especially for degradable resins, which are usually more expensive, the addition of plant fibers can yield PPCs with good mechanical performance, such as better toughness and elasticity of the materials, at a reduced cost [21,38,44]. Moreover, plant fibers would not affect the degradation performance of the products [45,46], which provides a new idea for the popularization and application of degradable resins.

#### 2.2.2. Additives

The surface of plant fiber is rich in hydroxyl and carbonyl, which makes plant fiber hydrophilic [24]. However, synthetic resins are mostly nonpolar structures resulting in poor compatibility between the two phases when they are blended with plant fibers [26,33,34], manifested by peeling between two phases, material strength decrease, and poor processability [7]. To increase the compatibility and improve the performance of PPCs, it is usually necessary to introduce proper functional groups for the surface modification of plant fiber to reduce the hydrophilicity, or use additives such as plasticizers and compatibilizers in the compounding process [4,6].

##### Surface Modification of Plant Fiber

Silane is a commonly used surface treatment agent. Cellulose can be treated with silanol aqueous solution, or silane coupling agents [47]. Olive husk flour [28], and a bamboo cellulose nanowhisker [47] treated with different silanes were all found to disperse more evenly in composites. Furthermore, the interfacial compatibility of PPCs was enhanced, and the mechanical properties and thermal stability properties were improved to varying degrees. However, an excessive amount of silanes would lead to its self-condensation reaction, which would cause insufficient silylation reaction and a lower grafting degree of the functional group [47].

Besides silane, other substances can also be used for the surface modification of plant fibers. Pyrrole can be oxidized and polymerized on the surface of bamboo fiber, and the resulting polypyrrole can improve the compatibility between bamboo fiber and PLA, and thus improve the mechanical properties and thermal stability of composites [48].

Alkali treatment is also a commonly used surface treatment method for plant fibers. Alkaline alkylation reaction occurs on the treated fiber surface, which is beneficial to blending with synthetic resins [7]. However, alkali treatment may reduce the inherent strength of plant fibers [47,49]. In a study, palm fiber (Macaíba) was first treated with maleic anhydride, sodium hydroxide or (3-methacryloxypropyl) trimethoxysilane (CAS: 2530-85-0), and then blended with polycaprolactone (PCL) [50]. The effect of this blend on mechanical properties of the composites was investigated, which showed that PPC with maleic anhydride-modified fiber had the best mechanical property, while sodium hydroxide had the worst modification effect.

##### Compatibilizer

Compatibilizers are often used to improve the properties of PPCs. Maleic anhydride, as a common reactive compatibilizer, can undergo esterification reaction with hydroxyl groups on the fiber surface, thus enhancing adhesion power between plant fiber and synthetic resin and improving the mechanical property of materials [7,51]. Compared with common PLA, adding 0.3% maleic-anhydride-grafted-PLA as a compatibilizer can improve the mechanical properties and waterproof performance of wood fiber/PLA composites [52]. Similar results were reported for the composite of bamboo fiber and PP using maleic-anhydride-grafted-PP as compatibilizer [22], and corn straw powder/low-density polyethylene (LDPE) composite compatibilized by maleic-anhydride-grafted-PE [53]. Lignin has also been reported as a coupling agent to increase the compatibility between plant fiber and plastic matrix, thus improving the mechanical properties of composites [42].

##### Other Additives

Additives commonly used in PPCs also include plasticizers, water and oil repellent, filling agent, nucleator, etc. [54]. Commonly used plasticizers include glycerol, ethylene glycol, urea, aliphatic acid, sugar alcohol, etc. [7]. Coffee silverskin/PHBV composites plasticized by acetyl tributyl citrate (ATBC) showed a better processability [39]. In the study of additives used in tableware made of ramie sticks, 3% liquid paraffin was found to bring a better waterproof effect, while lime carbonate, talcum powder, and white clay as composite filling agents could make the tableware have the strongest oil resistance [55]. Nano-silica is a common nucleator, which was reported to enhance mechanical properties, water resistance, and thermal stability of bamboo fiber/PLA composites when added up to 1.5% [56].

The functions and corresponding types of additives commonly used in current PPCs and products for food contact are summarized in Table 2.

## 3. Potential Hazards and Possible Sources

Like other food contact materials and products, the components of PPCs may move into the food through migration and diffusion via direct contact [57], thus causing safety problems. It is of great significance to analyze the components and sources of potential hazards for the safety assessment and risk control of PPCs. Due to the limited literature on migration data, the potential hazards of PPCs were speculated based on their possible ingredients. Hazards in PPCs may come from plant components, synthetic resins, additives, pesticide residues, or microorganisms, of which the potential safety risk of plant fibers is the key distinction between PPCs and common plastic materials.

### 3.1. Plant Ingredients

Apart from dominant ingredients with high molecular weight and stable structure, such as cellulose, hemicellulose, and lignin, plant fiber also contains many bioactive components, such as protein, polysaccharides, aldehydes, and ketones [24]. The composition of plant fiber has strong species specificity, which may also be affected by the place of origin and climate in which the plant is grown, for example, more terpenoids are needed for plants under greater environmental stress [40].

Some species of plants will produce toxins or allergens during their growth, which is one of the self-defense mechanisms of plants in long-term evolution [58]. Rosaceae plants will produce amygdalin in their seeds, which will be metabolized into highly toxic cyanide in the body after ingestion [59]. Flax contains linamarin and lotaustralin, that also belong to Cyanogenic Glycosides and can be hydrolyzed into cyanide under acidic conditions [60]. Cyanide will affect the utilization of oxygen in mitochondria and cause poisoning or death of the body. Ricin, a highly toxic and water-soluble protein, is contained in the seeds of castor oil plants and can cause serious symptoms such as gastrointestinal bleeding with a small amount [61]. Some studies have shown that lacquer sap from lacquer trees, natural rubber from *Hevea brasiliensis* tree, rice straw, and wood flour can cause allergic reactions such as contact dermatitis and asthma [62,63,64]. These substances may be removed during fiber processing, but they may also exist in some roughly processed plant powders.

### 3.2. Synthetic Resins

The safety risks introduced by synthetic resins mainly come from residual monomers, polymer decomposition products, oligomers, etc. MF resin, as a commonly used thermoset material [65], has been blended with plant fibers to produce tableware in many applications. However, MF resin will decompose under acidic conditions or high temperatures, resulting in the migration of melamine and formaldehyde [66]. Formaldehyde residues were tested in food contact materials made of various fiber/MF composites [57]. The migration of 25 volatile and semi-volatile substances, and 12 non-volatile substances have been found in bamboo/MF food contact materials, of which non-volatile substances were mainly melamine and its derivatives [8]. Federal Office of Consumer Protection and Food Safety of Germany (BMEL) randomly inspected 56 kinds of products on the German market and found that 11% of bamboo powder or corn starch tableware samples had excessive formaldehyde migration and 25% of samples had excessive melamine migration [67].

Microplastics would be another safety issue related to synthetic resins. These tiny particles were found to have many negative health effects, such as bio-accumulating, cytotoxicity, and reproductive toxicity, etc. [68,69]. Studies about polyethylene terephthalate (PET) water bottles, PET/nylon tea bags and PP feeding bottles [70] have reported high levels of microplastics release, which highlighted the risk of releasing microplastics directly from food contact materials.

### 3.3. Additives

Additives, with relatively low molecular weight and high reactivity, are easier to migrate and may have higher safety risks. Long-term exposure of maleic anhydride, which is commonly used in PPCs, will cause certain damage to the respiratory system, digestive system, and kidney [71,72]. Many countries and regions have also set a migration limit for this substance [73,74]. The migration of phthalates as a plasticizer, benzophenonone (BP) and 4-methylbenzophenonone (4MBP), which may be photoinitiators from photo-cured printing inks or adhesives, were also found in plant fiber-based materials [67,75].

In addition, the persistent organic contaminants perfluorooctane sulfonate (PFOS) and perfluorooctanoic acid (PFOA) that are refractory with long half-lives and have accumulation effects in organisms, can be used in plant fiber-based materials as surfactants for water-proof and oil-proof functions [76]. Relevant studies have shown that such substances may have reproductive and developmental toxicity and are related to cancer and thyroid diseases [77,78]. Thus, the possibility of perfluorinated or polyfluorinated substances migration should also be considered to avoid associated risks.

### 3.4. Other Hazards Plant Fiber May Introduce

Hazards of PPCs may also come from pesticide residues, antimildew agents, heavy metals or microorganisms. Plants, especially wheat, corn, and other crops, are susceptible to diseases and insect pests during their growth, and thus a large number of pesticides are needed. Antimildew agents and insecticides are also used during the storage process [7], resulting in the residues of the above substances in plant fibers. An inspection of disposable plant-based food contact materials conducted by BEUC, the European Consumer Organisation, reported a variety of insecticides residues, including some carcinogenic, teratogenic, mutagenic (CMR) substances, and endocrine disruptors [79].

Some plants will accumulate heavy metals during their growth, and these plants are often used for the treatment of contaminated soil [80]. Studies have shown that peanut shells have strong bioaccumulation ability for Cr and Pb [81]. Wetland plants, such as reeds have obvious adsorption effects on Cd, Cr, Cu, and other heavy metals [82]. Sugarcane has a higher bioconcentration factor for metal ions such as Mg, Cr, and Cd [83]. The heavy metals in plants will become part of the risks when these plants are made into food contact products.

Aflatoxin, produced by *Aspergillus flavus* and *Aspergillus parasiticus*, is a Group 1 human carcinogen with hepatotoxicity and carcinogenicity, and its intake is related to the incidence of liver cancer in the population [84]. Many studies have shown that peanuts, including peanut shells, are susceptible to aflatoxins contamination during growth and storage [81,85,86]. As aflatoxin has high thermal stability, the heat processing process can not destroy its activity [81]. Therefore, it is difficult to remove once it remains in the plant fiber. It is important to note that in order to control the growth of mold, besides optimizing the storage conditions, antimildew agents are often preferred and thus become a potential hazard in plant fibers.

## 4. Risk Management Measures in Countries around the World

Many countries in the world have established corresponding laws and regulations for the safety management of food contact materials to ensure food safety and public health. As PPCs have been widely used in food contact materials and products at present, some countries and regions have formulated relevant safety requirements for such materials (Table 3).

### 4.1. European Union

The European Union (EU) has established a relatively complete regulatory system for the safety management of food contact materials, which mainly adopts the combination of EU regulations, member states regulations, and Council of Europe (CoE) resolutions at the official level [87]. For food contact materials that have established EU regulations, such as Commission Regulation (EU) No 10/2011 for plastics [74], all member states are required to comply with the requirements of EU regulations. For food contact materials that have not yet established EU regulations, corresponding laws or regulations can be established by each member state. In addition, the Council of Europe has also formulated a series of guidelines for food contact materials, such as Res AP (2004) 4 for rubber products [88] and Res AP (2004) 1 for coatings intended for food contact use [89], which have important guiding significance for the safe production of corresponding materials.

In the EU, plastic materials and products added with plant fibers are treated as plastic materials and should comply with the relevant provisions of Commission Regulation (EU) No 10/2011. This regulation stipulates the safety requirements that plastic materials should meet and the list of substances allowed to be used, in which it is stated that “wood flour and fibers, untreated” and “ground sunflower seed hulls” can be used as additives in the production of plastic materials and products for food contact. However, there is no migration limit or quality specification for the above two kinds of plant fibers [74]. Currently, based on the discovered safety risks of PPCs, the European Commission has requested the European Food Safety Authority (EFSA) to re-evaluate the safety of “wood flour and fibers, untreated” as additives. Since the components in plant-derived materials are closely related to plant species and the processing process, EFSA Panel on Food Contact Materials, Enzymes and Processing Aids (CEP) holds that all plant materials to be used in plastic as additives should be evaluated for their safety case by case [90].

### 4.2. USA

Substances that may migrate into food from food contact materials and products are regarded as indirect food additives in the United States, and diversified management methods are adopted for their safety management, including Title 21 of The Code of Federal Regulations (21 CFR), Food Contact Notification (FCN), Threshold of Regulation (TOR), Generally Recognized as Safe (GRAS), and Prior-sanctioned Substances, etc. The relevant regulations on food contact materials and products and the list of approved substances are included in 21CFR [87].

Resins and additives used in plastics shall comply with the provisions of relevant sections of 21CFR. Substances not listed in 21CFR shall be approved by FCN procedure before they can be used in the production of food contact materials. However, the United States has not established special regulations or standards for food contact PPCs. Only in 21 CFR Section 177.1460 “Melamine-formaldehyde resins in molded articles” [91] and Section 177.1900 “Urea-formaldehyde resins in molded articles” [92], it is stipulated that the above two types of resins can be mixed with refined wood pulp to produce food contact materials and products. Adjuvant substances and limits of the chloroform-soluble extractives for the final product were also prescribed.

In addition, the FCN procedure has also approved the production and use of similar products, such as wooden trays used in the cooling process for short-term contact with food at low temperatures [93].

### 4.3. Japan

Before 2020, Japan mainly managed the safety of food contact materials through Notification No.370 “Specifications and Standards for Food, Food Additives, etc.” issued by the Ministry of Health and Welfare [94]. The third chapter of the notification stipulated the safety indicators and inspection methods of glass, ceramics and enamel, synthetic resin, rubber, metal, and other food contact materials. However, the list of raw materials for organic polymer materials such as synthetic resin and rubber was not specified in the notification, which allowed industry associations, such as Japan Hygienic Olefin and Styrene Plastics Association (JHOSPA), Japan Hygienic PVC Association (JHPA), and Japan Hygienic Association of Vinylidene Chloride (JHAVDC) to develop positive lists (PLs) of permitted substances to guide companies in their production [87].

In 2018, the revised Food Hygiene Law stipulated that synthetic resin in packaging material should adopt the management mode of PL, and only substances that have passed the safety assessment can be included in the list. Based on the PLs developed by relevant industry associations, the Ministry of Health and Welfare revised Notification No.370 and formulated the PL of food utensils, containers, and packaging (UCP), listing the types of polymers and additives allowed to be used in polymer materials such as plastics and coatings for food contact. The list allows “wood flour” and “natural fiber” to be used in plastics as additives, while specifying the application scope and maximum usage of the two additives [95]. The list has been officially implemented since June 2020.

### 4.4. China

China has established a series of mandatory national food safety standards, including general standards, product standards, inspection methods, and manufacturing process standards for the safety management of food contact materials. At present, there is no specific national food safety standard for food contact PPCs and its products. Such materials should refer to the requirements of GB 4806.7-2016 “National Food Safety Standard—Plastic Materials and Products for Food Contact” [96]. The use of resins and plant fibers should comply with GB 4806.6-2016 “National Food Safety Standard—Plastic Resin for Food Contact” [97], and GB 9685-2016 “National Food Safety Standard—Standards for the Use of Additives for Food Contact Materials and Products” [73], respectively.

China also formulated a series of non-mandatory product standards for plant fiber-based materials. GB/T 24398-2009 “Disposable Plant Fiber Chopsticks” describes the “plant fibers” as crop fibers (including rice straw, wheat straw, corn straw, bagasse, rice husk, peanut shell, etc.), bamboo fiber, wood fiber, etc., and stipulates the principled safety requirements for plant fibers, such as not deteriorating, mildewing, or being contaminated [98].

## 5. Conclusions

To date, PPCs have been widely used in construction, automobile, and other industries, with many advantages such as lightweight, low price, and degradability. However, there are still many safety problems to consider when they are applied to food contact materials.

First, according to the literature analysis, plant fiber itself may indeed introduce a variety of potential safety risks, including phytotoxins, allergenic proteins, microbial growth, heavy metals, and pesticide residues. These risks are usually ignored as plant fiber is declared to be “natural”. However, such safety risks are the key distinction between PPCs and traditional plastic materials and should be considered in risk assessment and risk management. At present, the management agencies in many countries have noticed the relevant problems and actively carried out countermeasures.

Secondly, due to the hydrophilic properties of plant fibers and the hydrophobic properties of synthetic resins, it is necessary to improve the compatibility of the two phases with various small molecule compounds when blending them together, and these small molecules are easy to migrate or diffuse into food. It is still unknown whether the compatibility between plant fibers and synthetic resins will decrease again, leading to the separation of the two phases, as the additive substances continue to migrate. In addition, the plant fiber will swell when it absorbs water and return to its original state again after dehydration. As a result, in the case of long-term and repeated contact with food, this phenomenon may also affect the compatibility between the two phases and then affect the overall migration of PPCs.

Thirdly, at present, most countries in the world generally regard plant fiber as the filling agent of plastic materials during product management. However, with technical renovation, some products with plant fibers as the main matrix have appeared, and the proportion of synthetic resin is very low. These products have a certain intersection with paper products (such as molded products of plant pulp), or bamboo and wood products (such as cork). Plant fiber has only certain plasticity, but it can still be called a “plastic” material in broad categories. However, further research is needed on their safety management, i.e., whether it is suitable to adopt exclusively the management of plastic materials, or whether such materials should be classified and differently managed.

Finally, the studies on PPCs primarily focus on mechanical performance and pay little attention to the safety risks. There is still a lack of relevant studies on the techniques of migration tests, the applicability of food simulants, migration patterns and mechanisms of hazards, systematic risk assessment methods, and safety requirements of plant fibers for these food contact composites. To protect the safety and health of consumers, traditional natural materials such as plant fiber still need further systematic studies.

## Figures and Tables

**Table 1:** Two categories of plastic/plant-fiber composites (PPCs) according to composition and processing technology.

Categories	Plant Fiber Content	Processing Technology	Coatings	Food Contact Application Scenario
PPCs with relatively higher plant fibers content	Up to 90%	Dry molding or wet compression molding process	Usually necessary	Disposable tableware
PPCs with relatively higher plastic resins content	30~70%	Extrusion molding	Not necessary	Reusable tableware

**Table 2:** Common additives for PPCs and products for food contact.

Function of Additives	Common Types of Additives
Fiber surface modification agent	Silanes, pyrrole, alkali (sodium hydroxide, etc.), maleic anhydride
Compatibilizer	Maleic anhydride, lignin
Plasticizer	Glycerol, ethylene glycol, urea, fatty acid, sugar alcohol, acetyl tributyl citrate (ATBC)
Waterproof and oil-proof agent	Liquid paraffin wax
Filler	Calcium carbonate, talcum powder, kaolinite
Nucleator	Nano-silica

**Table 3:** Safety Management of Plant Fiber/Plastic Composite Materials and Products for Food Contact.

Country or Region	Management Mode	Types of Allowed Plant Fibers	Safety Requirements of Plant Fiber	End Product Safety Requirements
EU	No proprietary regulation; Accordance with plastic materials and products; Positive list (PL) for raw materials	Wood flour and fibers, untreated; Ground sunflower seed hulls	Principle safety requirements	Commission Regulation (EU) No 10/2011
USA	No proprietary regulation; Allowing certain resins to be blended with plant fibers; FCN procedure	Refined wood pulp, wood flour, etc.	Principle safety requirements	21CFR; FCN
Japan	No proprietary regulation; Accordance with polymer materials; PL for raw materials	Wood flour; Natural fiber	Principle safety requirements	PL in Notification No.370
China	No proprietary regulation; Accordance with plastic materials and products; PL for raw materials	Comply with the provisions of GB 9685 standard	Principle safety requirements	GB 4806.7 standard

## Data Availability

Not applicable.
